# Genome Sequence of *Bacillus licheniformis* CGMCC3963, a Stress-Resistant Strain Isolated in a Chinese Traditional Solid-State Liquor-Making Process

**DOI:** 10.1128/genomeA.00060-12

**Published:** 2013-02-07

**Authors:** Qun Wu, Suqin Peng, Yao Yu, Yixue Li, Yan Xu

**Affiliations:** a State Key Laboratory of Food Science and Technology, Key Laboratory of Industrial Biotechnology of Ministry of Education, School of Biotechnology, Jiangnan University, Wuxi, China; b Key Laboratory of Systems Biology/Key Laboratory of Synthetic Biology, Shanghai Institutes for Biological Sciences, Chinese Academy of Sciences, Shanghai, China

## Abstract

*Bacillus licheniformis* CGMCC3963 is an important mao-tai flavor-producing strain. It was isolated from the starter (*Daqu*) of a Chinese mao-tai-flavor liquor fermentation process with solid-state fermentation. We report its genome of 4,525,096 bp here. Many potential insertion genes that are responsible for the unique properties of *B. licheniformis* CGMCC3963 in mao-tai-flavor liquor production were identified.

## GENOME ANNOUNCEMENT

Chinese mao-tai-flavor liquor is the symbolic drink of China, just as whisky is of Scotland and brandy is of France (1). It is produced through a complicated spontaneous and solid-state fermentation process, which is subjected to an extremely severe environment, such as high temperatures with acidic and ethanol stresses. It accumulates a distinctive microbial community with specific physiological properties and performance. *Bacillus licheniformis* plays an important role in liquor making, and it also represents one of the major populations of the microbial community (2).

A strain of *B. licheniformis* CGMCC3963 was isolated in this process. It was able to survive under severe conditions, including exposure to acids and ethanol, high temperature, and low water activity. It could also produce specific flavor compounds, including 2,3-butanediol, 3-hydroxy-2-butanone, 2-methylpropionic acid, 3-methylbutanoic acid, furaneol, maltol, and pyrazine (2). Such specific properties are distinct from those of the soil-dwelling type strains *B. licheniformis* 14580 and *B. licheniformis* DSM13 (3, 4).

In order to investigate its unique adaptations to various unfavorable conditions and its capacity to generate specific flavors, the genome of *B. licheniformis* CGMCC3963 was sequenced by a shotgun strategy using Illumina HiSeq 2000. This strategy produced 888 Mb of paired-end data with about 200-fold coverage of the genome. A total of 4,160,727 filtered paired-end reads were assembled into 205 contigs using Velvet v.1.0.14. Open reading frames (ORFs) were identified by Glimmer 3 (5) and were annotated using public databases, including the NCBI nonredundant (NR) database, Swiss-Prot, and Tremble. tRNA and rRNA genes were identified by tRNAScan (6) and RNAmmer (7). The alignment of the assembled genome and reference genomes was performed using Mummer (http://mummer.sourceforge.net/manual/).

The sequence of *B. licheniformis* CGMCC3963 is composed of a 4,525,096-bp circular chromosome, which is approximately 302,760 bp larger than that of *B. licheniformis* ATCC 14580. The G+C content is 45.21%. The chromosome contains 4,466 ORFs, 67 tRNA genes, and 10 rRNA operons, which together constitute about 91.35% of the genome. There are 411 unique genes present in this strain compared to *B. licheniformis* ATCC 14580. These unique sequences are important for the ability of this strain to survive under severe conditions and to produce specific flavor compounds.

### Nucleotide sequence accession numbers.

This Whole Genome Shotgun project has been deposited at DDBJ/EMBL/GenBank under the accession no. AMWQ00000000. The version described in this article is the first version, AMWQ01000000.
